# Comprehensive *in silico* analyses of flavonoids elucidating the drug properties against kidney disease by targeting AIM2

**DOI:** 10.1371/journal.pone.0285965

**Published:** 2023-05-18

**Authors:** Mahmoud Kandeel, Muhammad Nasir Iqbal, Iqra Ali, Saima Malik, Abbeha Malik, Sheikh Arslan Sehgal

**Affiliations:** 1 Department of Biomedical Sciences, College of Veterinary Medicine, King Faisal University, Al-Hofuf, Al-Ahsa, Saudi Arabia; 2 Department of Pharmacology, Faculty of Veterinary Medicine, Kafrelshikh University, Kafrelshikh, Egypt; 3 Department of Bioinformatics, The Islamia University of Bahawalpur, Bahawalpur, Pakistan; 4 Department of Biosciences, COMSATS University Islamabad, Islamabad Campus, Islamabad, Pakistan; 5 Department of Biochemistry, Abdul Wali Khan University Mardan, Mardan, Pakistan; 6 Department of Bioinformatics, University of Okara, Okara, Pakistan; Government College University Faisalabad, PAKISTAN

## Abstract

Kidney disorders are among the most common diseases and there is a scarcity of effective treatments for chronic kidney disease. There has been a progressive improvement in specific flavonoids for protective effects against kidney diseases. Flavonoids inhibit the regulatory enzymes to control inflammation-related diseases. In the present study, a hybrid approach of molecular docking analyses and molecular dynamic simulation was followed by principal component analyses and a dynamics cross-correlation matrix. In the present study, the top-ranked five flavonoids were reported, and the maximum binding affinity was observed against AIM2. Molecular docking analyses revealed that Glu_186, Phe_187, Lys_245, Glu_248, Ile_263, and Asn_265 are potent residues against AIM2 for ligand–receptor interactions. Extensive *in silico* analyses suggested that procyanidin is a potential molecule against AIM2. Moreover, the site-directed mutagenesis for the reported interacting residues of AIM2 could be important for further *in vitro* analyses. The observed novel results based on extensive computational analyses may be significant for potential drug design against renal disorders by targeting AIM2.

## Introduction

Inflammasomes are cytosolic receptors of the innate immune system that are responsible for the protection and activation of inflammatory responses against danger signals [1]. The inflammasomes consist of an upstream sensor protein, the apoptosis-associated speck-like protein containing a CARD (ASC) adaptor protein, and a downstream effector protein [2]. The inflammasomes are activated by distinct kinds of cytosolic pattern recognition receptors (PRRs) classified based on structural characterization to recognize cytosolic and nuclear pathogens. The activated inflammasomes further activate the caspase-1 and the activated protein induces inflammation and responds to harmful factors in the body. It further causes cell pyroptosis, and apoptosis regulates cellular pathways and plays a critical role in the innate immune system [3].

The activation of the inflammasome is the primary innate immune event that occurs in the host associated with several inflammatory disorders and plays a vital role in the pathogenesis of kidney disorders. The inflammasomes are also linked to a variety of microbial and non-microbial diseases including cardiovascular disorders, asthma, cancer, diabetes, Alzheimer’s, and atherosclerosis that affect the heart, intestine, lungs, and liver [4]. Moreover, it also performs an important role in autoimmune disorders such as psoriasis by recognizing host DNA [5]. However, kidney diseases recently gained increasing attention [1]. Kidney diseases have major and growing health issues worldwide. The global prevalence of kidney disease is estimated to be 8–16% while the healthcare cost for the treatment of kidney disease exceeds $130 billion [6, 7]. The innate immune system is typically implicated in the initiation and spread of inflammation in the kidneys. Inflammation plays an essential role in the pathogenesis and development of chronic kidney diseases. Furthermore, it has a prominent role in initiating renal fibrosis [8]. The inflammation can delay the capacity of the kidney to filter the surplus water and waste materials. Kidney inflammation is a serious and life-threatening condition that can result in chronic kidney disease [9]. The inflammasome is used as possible therapeutic target for aseveralrenal diseases.

The inflammasomes are divided based on structure as NLRP1, IPAF, NOD-, LRR-, NLRC4, NLRP3, and AIM2 inflammasomes. Interferon inducible protein 2 (AIM2) is a non-NLR protein expressed in the kidney and extensively characterized regarding renal diseases. AIM2 is a significant inflammasome component that belongs to the PYHIN family. AIM2 is a positively charged HIN domain that binds to cytoplasmic dsDNA through electrostatic interactions and pyrin (PYD)the at N terminal. The protein-protein interaction is responsible for the downstream activation of the adapter protein ASC to promote pyroptotic cell death in cells containing caspase-1 [10, 11]. AIM2 inflammasome is linked to kidney disease and plays a key role in regulating renal injury, inflammation, and fibrosis by assembling the multiprotein platforms for caspase activation [1]. In the absence of dsDNA, the interaction of PYD and HIN domains keeps the receptor autoinhibited. In the presence of cytosolic DNA, inhibition of AIM2 reduces inflammasome activation and leads to reduce inflammation-related diseases such as renal kidney [12].

There is a progressive improvement in natural compounds and has the least side effects. The natural products are rapidly gaining success in the treatment of renal illnesses [13]. Flavonoids are a class of low molecular weight phenolic compounds and are becoming increasingly popular due to various positive health effects. Flavonoids have the ability to exert multiple biological properties including protection from kidney diseases and use in nutraceutical, pharmaceutical, medicinal, and cosmetic applications [14]. Flavonoids are anti-inflammatory secondary metabolites having a 15-carbon (C6-C3-C6) backbone structure. A wide variety of higher plants that have red, blue, or purple hues contain flavonoids, and are secondary metabolites with varying phenolic structures [15].

Extensive *in silico* analyses demonstrates molecular docking analyses and molecular dynamic simulations to reveal novel flavonoids against kidney diseases. Extensive literature review was performed, and flavonoids were screened by molecular docking analyses followed by molecular dynamic simulations against kidney diseases by targeting AIM2. The inclusive computational study may reveal the potent evidence for a reliable framework to assist researchers to design and develop the potential compounds.

## Materials and methods

### Protein preparation

The 3D structure of the selected protein (interferon inducible protein AIM2) having PDB ID 3RN2 [12] was retrieved from Protein Data Bank (PDB) [16]. MODELLER 9.25 [17] was used to predict the missing residues (140–146, 341–347). Swiss PDB Viewer [18] and RAMPAGE [19] were used to optimize and minimize the protein crystal structure.

### Molecular docking analyses and docking validation

The molecular docking analyses were performed by utilizing AutoDock Vina [20] and flavonoids were used as ligands. The 2D structures of flavonoids were generated and minimized. High throughput virtual screening was performed and the top ranked 37 compounds of flavonoids were screened. The energy dissipated was calculated through AutoDock Vina and protein-ligand interactions were analyzed by employing PyMOL [21]. The 2D binding interactions were analyzed by utilizing BIOVIA Discovery Studio [22].

The optimum scoring function was used for high throughput virtual screening to scrutinize the suitable candidates. The highest scoring functions were generated to screen the unidentified compounds by using a decoy dataset of inactive and active ligands. A Database of Useful Decoys Enhanced was employed to create the decoy dataset [23]. The SMILES of the decoys were utilized to generate 2D structures of the selected compounds through Data Warrior [24]. The selected target protein was docked against active and decoys compounds. The receiver operating characteristic curve (ROC curves) were employed to assess the reliability of the selected scoring functions and attribute to higher points for active ligands against inactive ligands [25]. A script written in R language is used to calculate ROC curve [26].

### Toxicity analyses

Drug likeness and ADMET (adsorption, distribution, metabolism, excretion, and toxicity) properties were calculated by using pkCSM [27] and QikProp [28]. The lead likeness properties, mutagenicity and carcinogenicity were calculated for all the selected compounds [29].

### Molecular dynamic simulation

Desmond, a software from Schrödinger LLC [30], was utilized to perform Molecular Dynamic (MD) simulations for 100 nano seconds (ns). In molecular dynamics simulation, the receptor-ligand docking was performed to calculate the rigid binding analyses of the selected compounds against target protein [31]. MD simulation analyses were performed to predict the ligand binding status in physiological milieu by incorporating Newton’s classical equation of motion [32, 33].

The selected proteins and ligands were optimization and minimized by utilizing Maestro’s Protein Preparation Wizard. The steric clashes, bad contacts and distorted geometries were removed. System Builder tool was employed to build the systems and TIP3P (Intermolecular Interaction Potential 3 Points Transferable), an orthorhombic box was used as solvent model having OPLS_2005 force field [34]. Counter ions were used to neutralize the models and added 0.15M sodium chloride to simulate physiological conditions with 300K temperature and 1 atm pressure throughout the simulation period. For inspection, trajectories were stored after every 100 pico seconds (ps) and protein-ligand stability was confirmed by Root Mean Square Deviation (RMSD) over time. The Principal Component Analysis (PCA) and dynamic cross-correlation matrix (DCCM) were analyzed by using Bio3D package of R [35] A script written in R language is used to calculate PCA and DCCM [36, 37].

### Molecular mechanics and generalized born surface area (MM-GBSA) calculations

The molecular mechanics generalized Born surface area (MM-GBSA) module of prime was used to determine the binding free energy (Gbind) of docked complex during MD simulations of AIM2 complexed with CID107876. Using the OPLS 2005 force field, VSGB solvent model, and rotamer search techniques, the binding free energy was estimated. The MD trajectory frames were chosen at intervals of 10 ns after the MD run. The total free energy binding was calculated using Eq 1:

dGbind=Gcomplex−(Gprotein+Gligand) (1)

Where, dGbind = binding free energy, Gcomplex = free energy of the complex, Gprotein = free energy of the target protein, and Gligand = free energy of the ligand.

## Results and discussion

**Fig 1:**
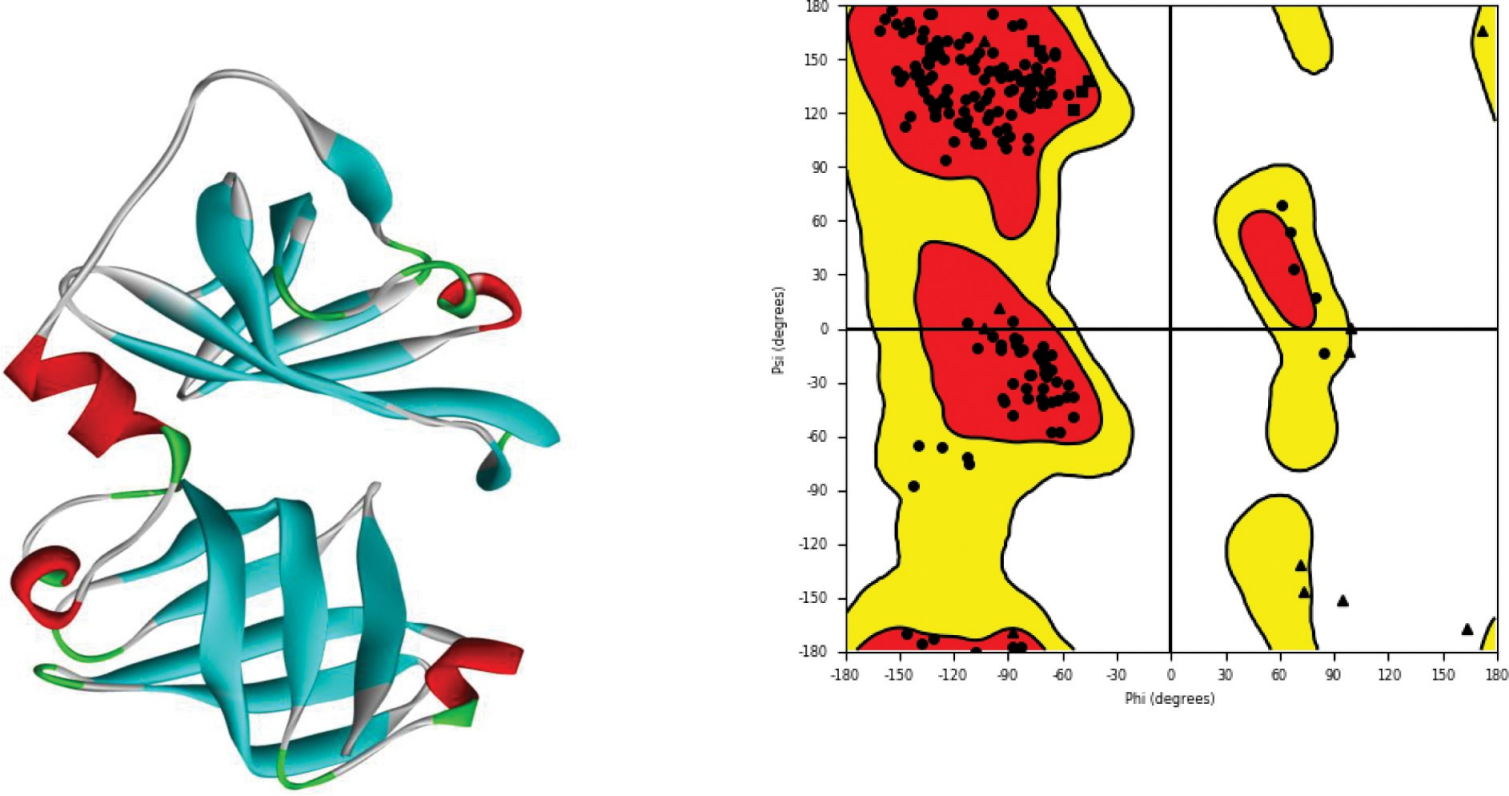
3D structure of the selected protein retrieved from PDB along with its Ramachandran plot displaying different sections of target protein structure.

The 3D structure of the target protein (3RN2) was retrieved from PDB. The total structural weight of the selected protein was 59.60 kDa. The global symmetry of the selected protein was cyclic-C2 and Homo 2-mer A2 was calculated as stoichiometry [12]. The missing residues from the selected protein structure were predicted, optimized, and minimized (Fig 1) [38] for further analyses. In this process proper bond order assigned, adequate hydrogen atoms added, and loop refinement of target protein was performed to get the native conformation of protein [39, 40]. The predicted structure was evaluated, and it was observed that the overall quality of the predicted structure was 99.23%. It was observed that the residues were displayed as circles, while glycine was plotted as triangles and proline as squares (Fig 1) [41].

The molecular docking analyses were performed for the top ranked 37 flavonoids. Before performing docking, we remove all water molecules and ions to get accurate results. The docking was performed with prepared protein target. The grid box was used with center_x = -12.3038, center_y = -3.8932, center_z = -23.6318, size_x = 62.0402586746, size_y = 44.1229688644, and size_z = 44.4926281977 dimensions [42]. The bioinformatics tools are of great significant and used on selected compounds for further refining of molecules based on ADMET properties resulting in identification of top ranked 5 compounds that satisfy the Lipinski’s rule of five and least binding energy (Table 1) [43].

**Table 1:** ADMET properties, binding affinity, and pharmacophore score of top compounds (mol_MW: Molecular Weight, donorHB: Hydrogen Bond Donor, accptHB: Hydrogen Bond Acceptor, QPlogPo/w: Predicted octanol/water partition coefficient, QPlogHERG: Predicted IC50 value for blockage of HERG K+ channels, QPPCaco: Predicted apparent Caco-2 cell permeability in nm/sec, QPlogBB Predicted brain/blood partition coefficient, QPlogKhsa: Prediction of binding to human serum albumin, and binding affinity from docking in kcal/mol).

PubChem ID	mol_MW	donorHB	accptHB	QPlogPo/w	QPlogHERG	QPPCaco	QPlogBB	QPlogKhsa	Binding Affinity (Kcal/mol)
**107876**	594.528	10	11.65	0.025	-6.156	83	-4.458	-0.437	-8.6
**370**	170.121	4	4.25	-0.585	-1.396	10.027	-1.659	-0.987	-5.7
**9064**	290.272	5	5.45	0.427	-4.813	51.696	-1.91	-0.43	-7.2
**72277**	306.271	6	6.2	-0.203	-4.524	21.067	-2.313	-0.56	-6.9
**5281855**	302.197	4	8	-1.306	-3.852	7.958	-2.396	-0.663	-7.9

The molecular weight of all the selected compounds was calculated and 130.0 to 725.0 (grams per mole) molecular weight was observed for all the selected compounds [44]. Thereafter, five top ranked compounds were analyzed on the basis of their binding affinity, ADMET properties and least binding energy values (Table 1) revealed that the scrutinized compounds showed significant biological properties. Hydrogen bond donor was calculated to share the electrons of solute to water molecules. In addition, the estimated amount of hydrogen bonds that a solute from water in aqua solutions would accept is known as a hydrogen bond acceptor. The non-integer values having recommended range of 0.0–6.0 were selected for hydrogen bond donor and acceptor. The selected values were as average across several different configurations. The utilized values were calculated as an average over multiple states leads to be non-integers and operates in between 2.0 and 20.0. The octanol water partition coefficient was calculated in between -2.0 to 6.5as the value of inhibitory concentration (IC50) for the blockage of HERG K+ channels. Caco2 cell permeability prediction was also calculated and observed reliable. The gut-blood barrier was also observed by using Caco2 cells. The values of the observed compounds ranges from 0 to 25, considered as poor however, >500 considered as reliable for further analyses. The expected brain and blood separation ratio was also calculated. The dopamine and serotonin were observed negative against Central Nervous System (CNS) as the selected compounds were polar in nature to cross the blood-brain barrier. The human serum albumin binding predicted, or QPlogKhsa, has a range of -1.5 to 1.5 [45].

**Fig 2:**
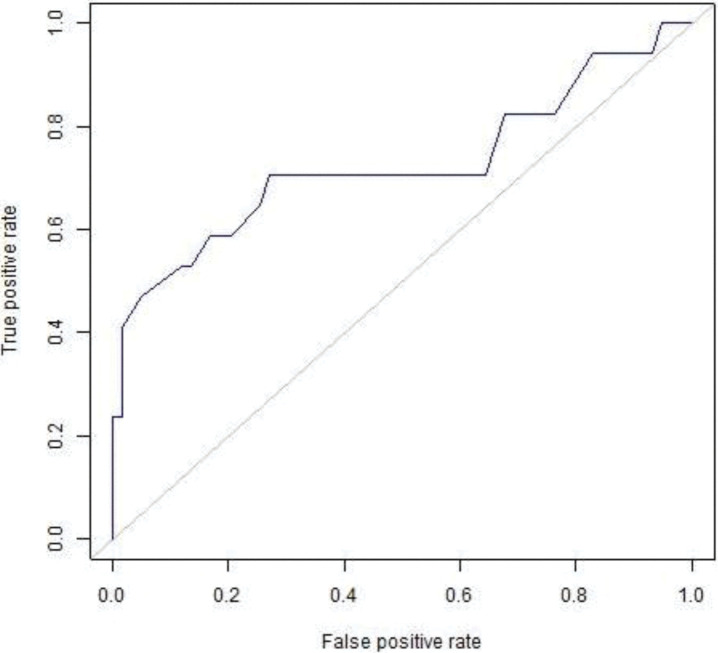
ROC curves of docking validation score.

The visual depictions of the relationship between the candidates of test specificity and sensitivity were calculated through ROC curves. The ROC curves were generated through graphing the percentage of genuine positives relative to the percentage of the false positives relative to the percentage of true negatives. The designed ROC curves pattern was utilized to verify the selected compounds for molecular docking analyses so the selected compounds should be from active ligands instead of inactive ligands (decoys). It was also observed that the designed pattern scrutinized the active ligands from top ranked compounds of the selected database. 0.7253 area was observed are under the curve (Fig 2) and enrichment factor was observed in top 1% (13.88) as reliable. ROC curve is the relationship between sensitivity and specificity. It represents true positive and false positive fractions on y-axis and x-axis, respectively. 0.7253 is a good area under the ROC which shows that the docking tool performs significantly accurate docking with the target protein and selected compounds compound [46].

**Fig 3:**
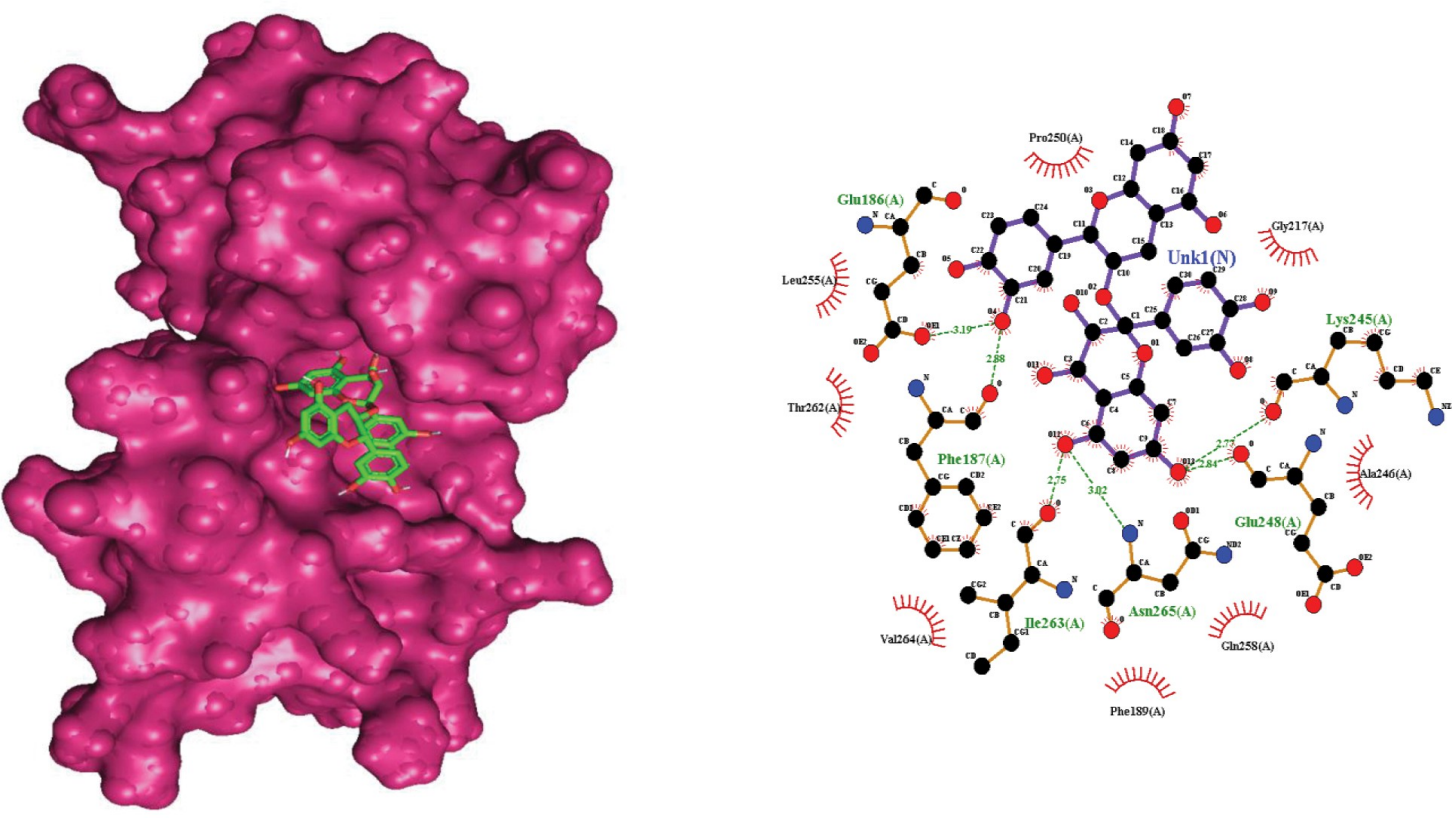
The interaction residues of the selected compound against the selected protein along with bond length.

Extensive analyses revealed that Procyanidin (ID:107876) was more efficient among the selected compounds. The scrutinized compound showed affective binding affinity and critical binding residues Glu_186, Phe_187, Lys_245, Glu_248, Ile_263, and Asn_265, observed in molecular docking analyses with AIM2 (Fig 3). MD simulation coupled with molecular docking analyses suggested that the selected compound must satisfy the drug properties having least binding energy. By satisfaction of the selected parameters of binding energy, binding affinity and ADMET properties, it is suggested that Procyanidin (ID:107876) is a potent compound against kidney disease by targeting AIM2 (Table 1) [47].

Catechin and epicatechin molecules combine to generate procyanidin as an oligomeric chemical. The de-polymerization in an oxidizing environment leads to produce cyanidin. Polyphenols is the largest class of secondary metabolites. Proanthocyanins is condensed tannins, precursor to procyanidins and type of polyphenol. Procyanidins are polyphenols prevalent in dietary fruits, vegetables, legumes, nuts, and grains and have a number of biological actions including chemo preventive [47, 48]. The optimal chemical complex with the protein target was simulated by using MD simulation analyses for 100 ns. RMSD and RMSF values were determined by means of MD trajectory analyses.

**Fig 4:**
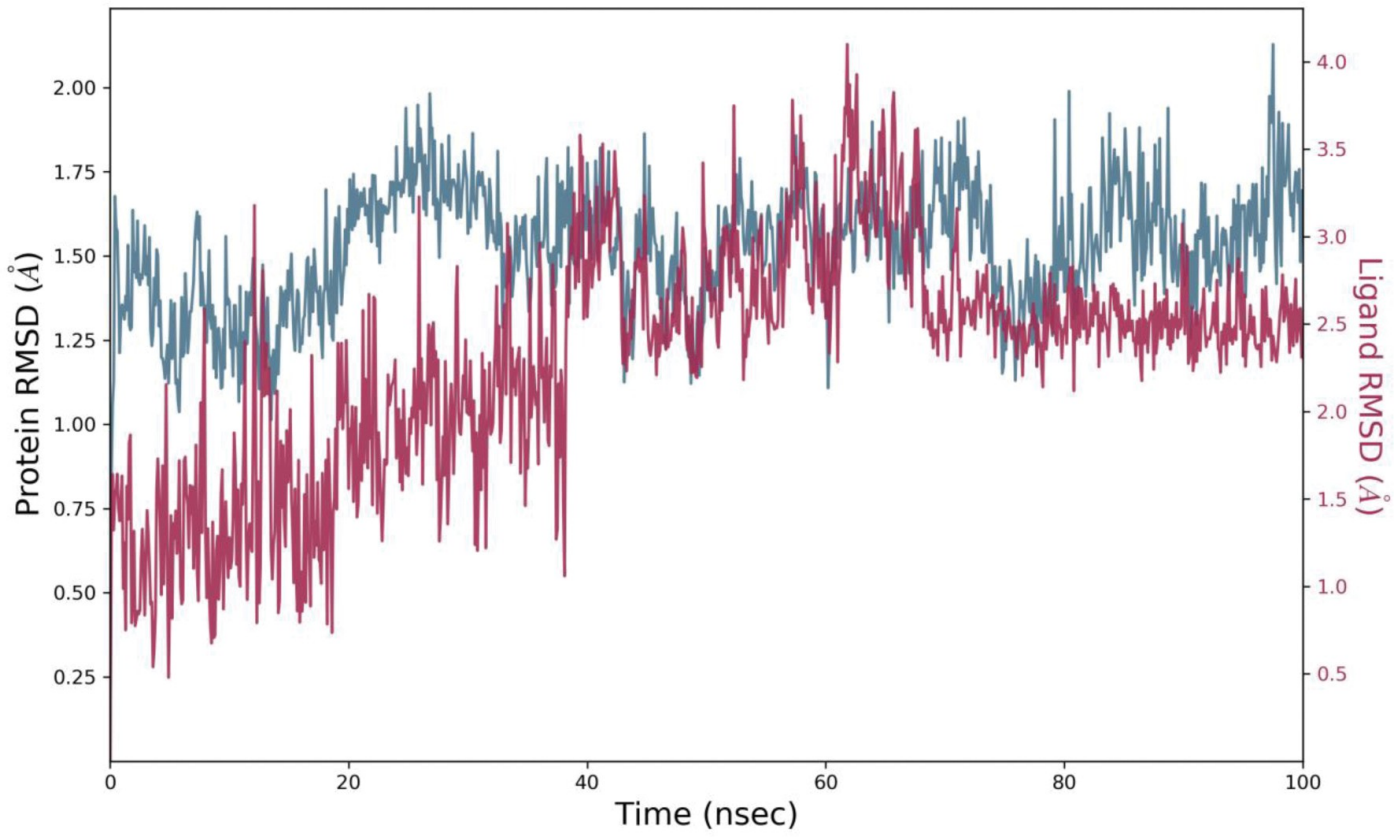
The variation in the root mean square deviation (RMSD) between the C-alpha atoms of proteins and ligand (107876-3RN2) over time. Protein RMSD shifts over time are plotted on the left Y axis. Differences in ligand root-mean-square deviation (RMSD) over time are plotted along the right Y-axis.

The time-dependent variation in RMSD values for C-alpha atoms in ligand-bound proteins showed the stability of the complex (Fig 4). The RMSD plot showed that the complex 107876-3RN2 stabilized at 10 ns. However, there was a slight increase in RMSD of protein bound ligand at 40 ns. This flip could be due to the conformational change in the rotatable bonds of ligand. The two-dimensional representation of ligand in (Fig 3) shows that it has some rotatable bonds. Torsion angle of ligand cause these types of flips [49]. After 40 ns, no obvious change and variation was observed throughout the simulation analyses of ligand fit on protein. It was observed that the average RMSD of protein structure (PDB ID: 3RN2) was 1.5235 with 0.1846 standard deviation. The average RMSD of ligand with respect to protein was 1.4198 with 0.79066 standard deviation. The RMSD showed variations however, no clear variation has been observed in the RMSD calculation of ligand after equilibrium. This showed that the ligand remained bound to the binding pocket of the protein [50, 51].

**Fig 5:**
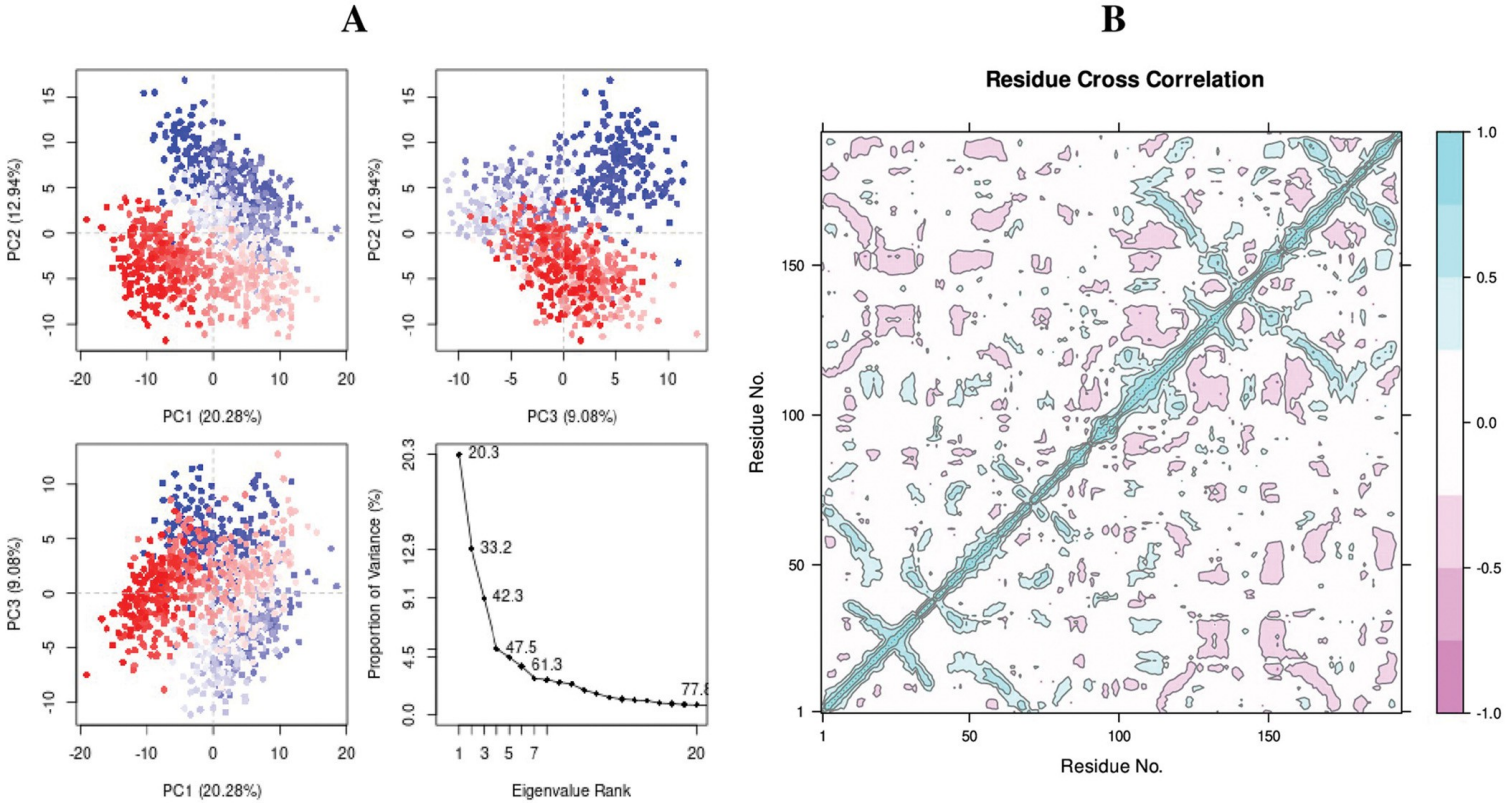
(A) Principal Component Analysis eigenvalue plotted versus the percentage of variance (107876-3RN2). The varying areas are displayed on three separate sections. Variations in PC1, PC2, and PC3 add up to 20.08 percent, 12.94 percent, and 9.08 percent, respectively, (B) Complex 107876-3RN2 dynamic cross-correlation map. The positive and negative correlations of the residues are depicted by cyan and purple colors respectively.

Protein dynamics are characterized by Principal Component Analysis (PCA) [52]. The observing collective trajectory motions during MD simulations analyses were calculated. The graph of eigenvalues (protein) against eigenvector index (eigenmode) for the first 20 modes of motion (rn2 = 107876) (Fig 5A) showed stability. The eigenvalues depicted the hyperspace eigenvector fluctuations. In simulations analyses the eigenvectors having higher eigenvalues regulates the total mobility of the target protein. The top five eigenvectors in utilized systems showed dominant movements and had larger eigenvalues (20.3–61.3%) than the other eigenvectors. All changes were observed and plotted in three PCs (PC1, PC2, and PC3). PC1 clusters had the largest variability (20.28%), PC2 showed variability (12.94%), and PC3 had the lowest variability (9.08%) (Fig 5A). As a result of its low variability, PC3 has a more compact structure than PC1 and PC2 and was considered as more stabilized protein ligand binding complex. The simple clustering in PC subspace revealed conformational variations across all the groups. The blue color exhibits the most significant mobility; white color indicates intermediate movement, and red indicating less flexibility [53].

The selected ligand (ID: 107876) and the target protein (3RN2) showed significant correlation through the high pairwise cross-correlation coefficient value on the cross-correlation map (Fig 5B). The magenta color represents anti-correlated residues (-0.4), whereas cyan color represents the correlated residues (>0.8). It was observed that the large number of pairwise correlated residues between the target protein (AIM2) and the selected ligand [54].

**Fig 6:**
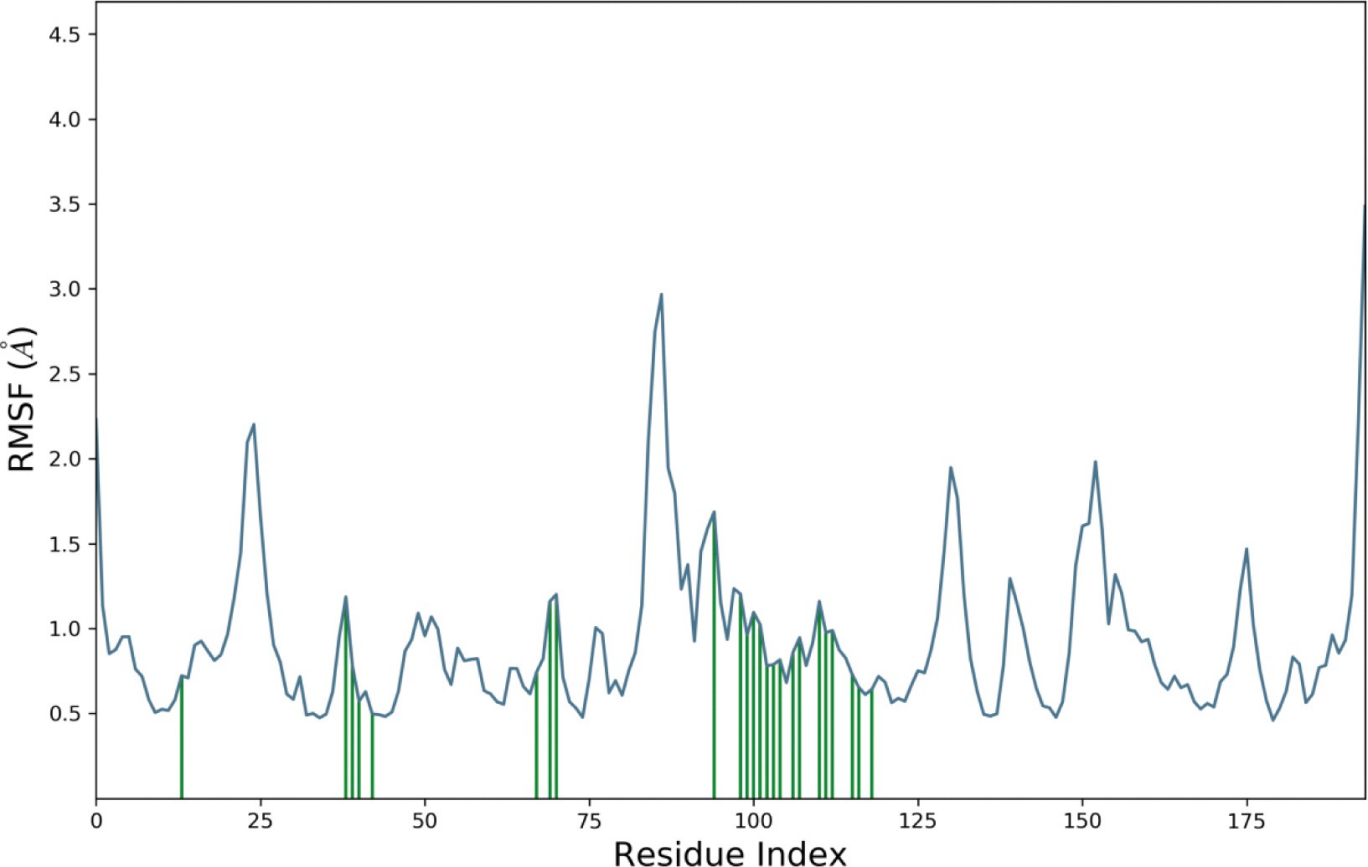
Root Mean Square Fluctuation (RMSF) of the target protein residues complexed with the selected ligand.

**Fig 7:**
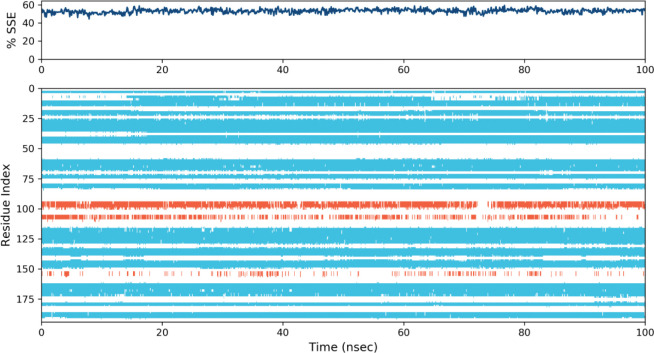
Elements of protein secondary structure are dispersed across protein-ligand complexes with respect to residue index. The alpha helices are represented by the red columns and the beta strands by the blue ones.

RMSF value of the protein-ligand complex was calculated (Fig 6). Based on MD trajectories, the higher peaks of the residues in loop regions, N- and C-terminal zones (Fig 7) showed the stability. The stability of the selected ligand binding against the target protein showed low RMSF values. The secondary structure features including alpha-helices and beta-strands were predicted throughout the simulation. Secondary Structure Elements graph was plotted against the residual index to calculate the distribution across the protein structure. It was observed that 3.98% was alpha helices, 49.14% of beta sheets 3.7% of remaining elements of the secondary structures and the total was observed as 53.13%. Ratio of alpha helices and beta sheets also affect the RMSD of protein. As there are rigid region of protein so the residues in these structures showed low RMSD as compared to the residues lies in coils and loops [55, 56].

**Fig 8:**
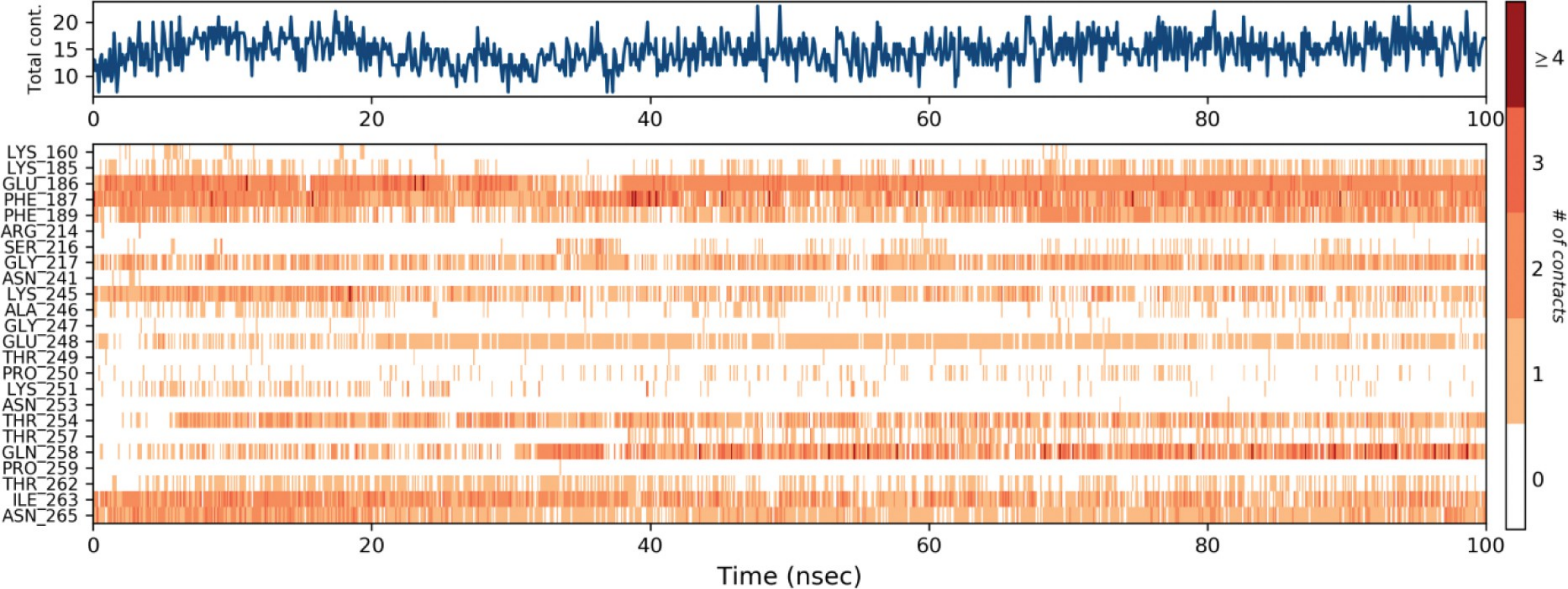
Protein-ligand contact heatmap throughout trajectory.

The hydrogen bonds constituted the vast majority of the significant ligand-protein interactions (Fig 8). The hydrogen bonding was observed for Glu-186, Phe-187, Glu-248, Gln-258, Ile-263 and Asn265 residues. The ligand-protein interaction was also critically observed over the course of the simulation analyses. The molecular contacts and interactions (H-bonds, hydrophobic, ionic, and water bridges) showed the interaction between the target protein and the selected ligand. Each frame of the trajectory was calculated at x-axis and the interaction of the ligand. Various independent interactions with the ligand were also observed (Fig 8) [57].

In present work, molecular docking analyses coupled with MD simulation were performed, and missing residues from the 3D structure of AIM2 was predicted. The simulated complexes showed a reliable degree of accuracy, specifically at the binding site of the target protein. Molecular docking analyses and MD simulation analyses revealed the interactional residues of the selected ligands and the receptor protein. The selected ligand showed least binding energy and critical binding residues Glu_186, Phe_187, Lys_245, Glu_248, Ile_263, and Asn_265, observed with AIM2. The reported compound showed least binding energy and efficient properties. Molecular docking analyses and MD simulation suggested that the efficient ligand must have affective binding affinity, reliable ADMET properties and least binding energy. In the light of selected parameters of least binding energy and ADMET properties, it is suggested that Procyanidin (ID:107876) are potential drug molecules. It stands to the reason that the reported ligand has the propensity to be potent ligand [58, 59].

The MMGBSA method is frequently used to evaluate the binding energy of ligands to protein molecules [60]. The influence of additional non-bonded interaction energies as well as the binding free energy of each AIM2-CID107876 complex were evaluated. The binding energy of the ligand CID107876 to AIM2 is -65.7268 kcal/mol. Gbind is governed by non-bonded interactions such as G_bind_Coulomb, G_bind_Packing, G_bind_H_bond_, G_bind_Lipo, and G_bind_vdW (Table 2). The S1 Table contains all the MM-GBSA results. The average binding energy was mainly influenced by the G_bind_vdW, G_bind_Lipo, and G_bind_Coulomb energies across all types of interactions. The GbindSolvGB and Gbind Covalent energies, on the other hand, made the smallest contributions to the final average binding energies. Additionally, AIM2-CID107876 complexes showed stable hydrogen bonds with amino acid residues by their G_bind_H_bond_ interaction values. As a result, the binding energy derived from the docking data was well justified by the MM-GBSA calculations that came from the MD simulation trajectories [61].

**Table 2:** Average MM-GBSA binding energy calculation of CID107876 with AIM2 after every 10 ns from MD Simulation trajectories.

Energies (Kcal/mol)	AIM2-107876
**dG**_**bind**_	-65.7268
**dG**_**bind**_ **Lipo**	-17.0467
**dG**_**bind**_ **vdW**	-42.1463
**dG**_**bind**_ **Coulomb**	-49.3653
**dG**_**bind**_ **H**_**bond**_	-5.07422
**dG**_**bind**_ **Packing**	-3.5666

## Conclusion

In conclusion, the current work suggested that the reported compound Procyanidin (ID:107876) are effective in kidney disease by targeting AIM2. Though extensive ***in silico*** analyses including molecular docking analyses and molecular dynamic analyses seem to be enough to conclude that Procyanidin (ID:107876) may be the potent option for kidney disease by targeting AIM2. The reported compound will be useful to researchers and may lead to the development of a new medicine for the treatment of renal inflammasomes.

## Supporting information

S1 Table MM-GBSA binding energy calculation of bonded and non-bonded interactions of CID107876 with AIM2 after every 10 ns from MD simulation trajectories. (CSV)
